# Supervised spatial classification of multispectral LiDAR data in urban areas

**DOI:** 10.1371/journal.pone.0206185

**Published:** 2018-10-24

**Authors:** Lian-Zhi Huo, Carlos Alberto Silva, Carine Klauberg, Midhun Mohan, Li-Jun Zhao, Ping Tang, Andrew Thomas Hudak

**Affiliations:** 1 Institute of Remote Sensing and Digital Earth, Chinese Academy of Sciences, Beijing, China; 2 Department of Natural Resources and Society, College of Natural Resources, University of Idaho, Moscow, ID, United States of America; 3 Biosciences Laboratory, NASA Goddard Space Flight Center, Greenbelt, MD, United States of America; 4 Department of Geographical Sciences, University of Maryland, College Park, Maryland, MD, United States of America; 5 Federal University of São João Del Rei–UFSJ, Sete Lagoas, MG, Brazil; 6 Department of Forestry and Environmental Resources, North Carolina State University, Raleigh, NC, United States of America; 7 US Forest Service (USDA), Rocky Mountain Research Station, RMRS, Moscow, ID, United States of America; Western University, CANADA

## Abstract

Multispectral LiDAR (light detection and ranging) data have been initially used for land cover classification. However, there are still high classification uncertainties, especially in urban areas, where objects are often mixed and confounded. This study investigated the efficiency of combining advanced statistical methods and LiDAR metrics derived from multispectral LiDAR data for improving land cover classification accuracy in urban areas. The study area is located in Oshawa, Ontario, Canada, on the Lake Ontario shoreline. Multispectral Optech Titan LiDAR data over the study area were acquired on 3 September 2014 in a single strip of 3 km^2^. Using the channels at 1,550 nm (C1), 1,064 nm (C2) and 532 nm (C3), LiDAR intensity data, normalized digital surface model (nDSM), pseudo normalized difference vegetation index (PseudoNDVI), morphological profiles (MP), and a novel hierarchical morphological profiles (HMP) were derived and used as features for the classification. A support vector machine classifier with a radial basis function (RBF) kernel was applied in the classification stage, where the optimal parameters for the classifier were selected by a grid search procedure. The combination of intensity, pseudoNDVI, nDSM and HMP resulted in the best land cover classification, with an overall accuracy of 93.28%.

## Introduction

Urban land cover mapping is important for urban land management and planning [1, 2]. Remote sensing technology, with a fast revisiting period and a large coverage, provides the primary source data for a better understanding of urban land cover [3]. To fully utilize the data, land cover maps are usually derived based on visual interpretation or automatic classification methods [4].

Regional and global urban areas are mainly monitored by mapping impervious surface areas, which have anthropogenic features through which water cannot infiltrate into the soil (e.g., roads, parking lots, and rooftops) [5]. Impervious surface areas have been monitored by various satellite sensors, such as the Operational Linescan System (OLS) from the Defense Meteorological Satellite Program (DMSP) that images nighttime light [6], 250–1000 m Moderate Resolution Imaging Spectroradiometer (MODIS) imagery [3,7], and 30 m Landsat imagery [8,9]. Even though the coarse to moderate spatial resolution of these image types describes the spatial extent of urban areas, they often are not able to resolve the extent and spatial arrangement of subtypes within urban areas (e.g., buildings, roads, and trees).

High spatial resolution imagery (e.g., IKONOS and QuickBird) provides new opportunities to map detailed urban areas at a fine scale by providing more detailed observations of the Earth [10, 11]. Nonetheless, urban land cover classification is still a challenging task due to the spectral heterogeneity and structural diversity of the complex geospatial objects present [12]. Significant efforts have been made to advance image classification efficiency by focusing on object based image analysis [13–15] extracting spatial features [16, 17] and even scene based image analysis methods [18]. However, the effects of shadowing and relief displacement still pose considerable challenges [19]. Hence, active sensors such as airborne LiDAR (i.e., Light Detection and Ranging) data have been investigated for land cover classification in urban areas in the last decade [20].

Airborne LiDAR provides the 3-D coordinates of the survey area by collecting a cloud of laser range measurements [20]. Based on 3-D point clouds, LiDAR can be further interpolated into raster layers of surfaces, such as digital surface models (DSM), and intensity data [21]. To fully utilize the LiDAR data for land cover classification, several groups of methods have been proposed [20, 22–26]. The first category of methods directly uses LiDAR point clouds, e.g., LiDAR point clouds can be directly used for urban feature extraction [23]; or semantic point cloud interpretation can be performed based on optimal neighborhood selection [24]. The second category of methods mainly relies on LiDAR points derived products, intensity image [25] or both intensity and DSM image (i.e., height information) [26]. The study [26] done by Zhou suggests that LiDAR data alone (by combining intensity and DSM data) could potentially be useful to accurately map urban land cover. Another popular category of methods adopts the strategy to combine LiDAR data with optical images (e.g., multispectral or hyperspectral images) for detailed urban land cover mapping [22, 25, 27, 28]; DSM data compliment multi-spectral information from passive optical imagery to identify different land cover types. A survey of LiDAR data for urban land cover mapping can be found in [20].

However, most of the previous studies exploiting LiDAR for urban land cover mapping used single-band LiDAR due to limited data availability. Since backscattered energy from LiDAR depends on target materials, target roughness, and laser wavelength [29], single-band LiDAR has a restricted ability to discriminate land cover types. Hence, multispectral LiDAR holds greater promise to map urban land cover classes. New multispectral LiDAR data sensors (e.g., Multispectral Optech Titan LiDAR), which measures backscattered energies at different wavelengths, provide new opportunities to classify urban land cover effectively [30]. Since the release of the first commercial airborne multispectral LiDAR system, several studies have been tested to assess capabilities to produce more accurate land cover maps [29–32, 33–37]. For instance, Teo et al. demonstrated that multi-wavelength LiDAR can provide higher accuracy than single-wavelength LiDAR for land cover classification [29]. Bakula et al. [35] applied a maximum likelihood classifier in a six-class land cover classification and achieved an overall accuracy of 90%. A maximum likelihood classifier was also used to classify intensity and height images, and the authors found that height information is important for urban land cover mapping [34]. Fernandez-Diaz et al. [36] assessed capabilities of the Titan multispectral LiDAR for land cover classification, bathymetric mapping and canopy characterization. Zou et al. [37] adopted the object-based method and found that pseudo normalized difference vegetation index (pseudoNDVI) calculated from multispectral LiDAR may improve vegetation identification. In another study [33], an object-based analysis was also performed on multispectral airborne LiDAR data for land cover classification and map updating in Finland.

Motivated by previous studies [29, 33–37], this research is focused on classification of the multispectral LiDAR intensity rasters, sinceraster format is a more convenient than point clouds for land cover mapping. Previous studies have shown that multispectral LiDAR performs better than single band LiDAR in land cover classification [29], and found pseudoNDVI [37,38] and DSM [34,35] to be useful for improving classification accuracy of multispectral LiDAR data. in the novelty of this studyis that we investigated the role of spatial information in improving urban land cover classification results employing multispectral LiDAR intensity data (i.e., spectral-spatial classification [39]).

Spectral-spatial classification aims to improve classification accuracy by combining spatial information contained in the multispectral or hyperspectral data [39], to produce more accurate classification maps [40]. It is widely studied with a rich set of algorithm developments [39,41–43], especially in hyperspectral image analysis. Among these methods, morphological profiles (MP) perform well due to their ability to capture spatial relationships among different land cover types [41,42]. In mathematical morphology [44], two fundamental operators are erosion and dilation, which are applied to an image with a set of known shapes, called the structuring elements (SE). Different combinations of erosion and dilation constitute opening and closing operations, which are the building blocks of MP. Functionally, the opening operation can remove objects smaller than the structuring elements, while the closing operation can fill small holes and connect adjacent objects. The traditional MP operates on the characteristic image (mainly the first or first several principal components) of multispectral or hyperspectral images. Basically, it is performed assuming the image data are in the same vertical level; i.e., the morphological operation is performed only considering its spatial neighborhood pixels while lacking the capability to consider whether its neighborhood pixels obviously belong to another land cover class (e.g., the opening and closing operation for a tree pixel bordering grass pixels will impose effects on those grass pixels). However, the LiDAR-derived nDSM provides the vertical context of the image, thus offering opportunities to introduce vertical context while extracting the MP feature. To take full advantage of the nDSM data, a novel hierarchical morphological profiles (HMP) feature is proposed. Hence, the specific goals of this study are as follows: (1) to assess the usefulness of the MP for improving multispectral LiDAR data classification; and (2) to further study effectiveness of the proposed HMP.

## Materials and methods

### Study area description

**Fig 1:**
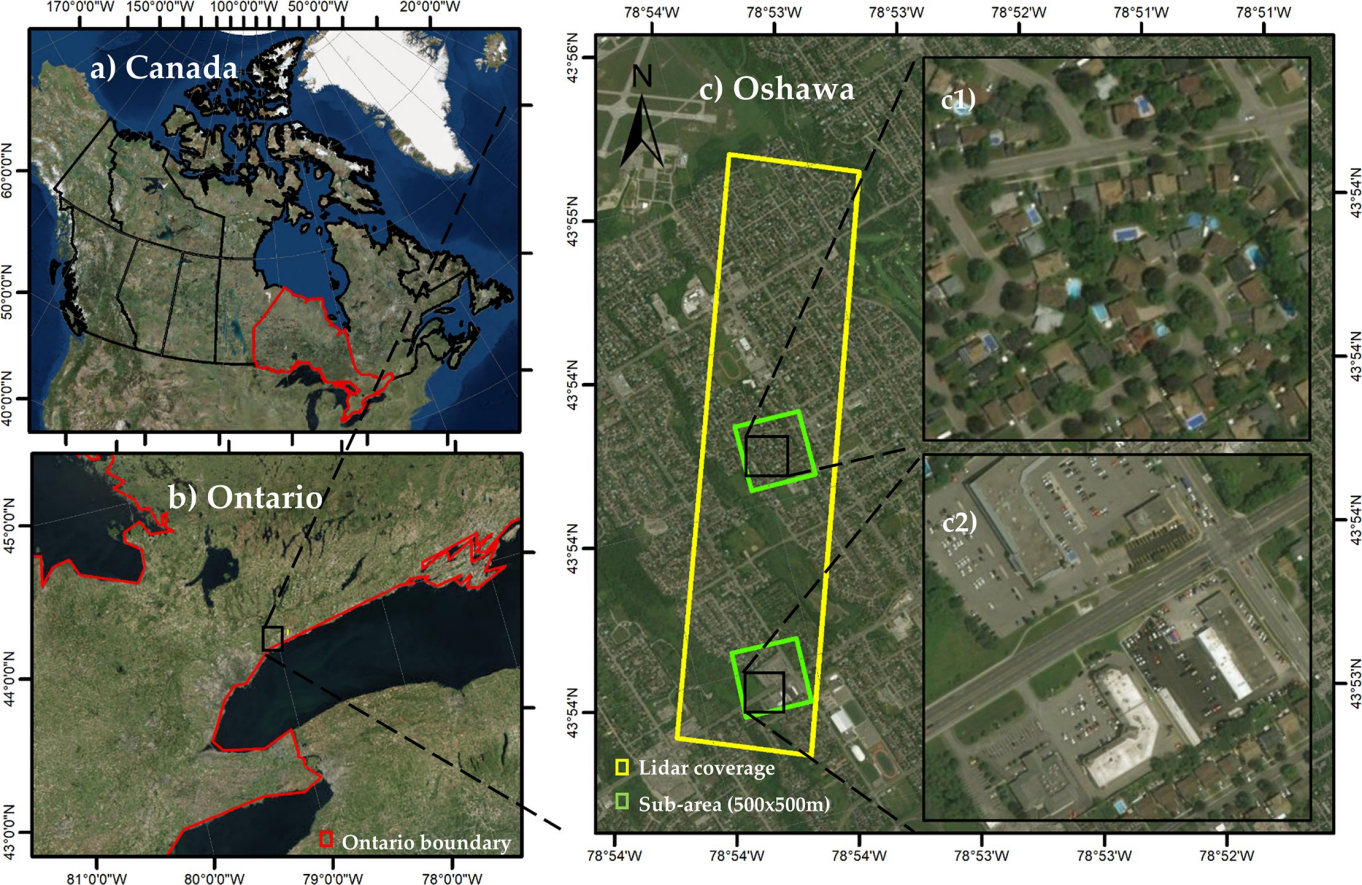
Study area location in A) Canada; B) State of Ontario; C) City of Oshawa, with subset areas 1) and 2) for visualizing the classification results.

The study area is located in Oshawa, Ontario, Canada, on the Lake Ontario shoreline (see Fig 1). Oshawa lies in Southern Ontario, approximately 60 kilometers east of Toronto. It is the largest municipally in the Regional Municipality of Durham, and it is commonly viewed as the eastern anchor of the Greater Toronto Area. Oshawa is a typical urban area with complex spatial assemblages of vegetation, buildings, roads, and other man-made features. The city presents a population density of 1,027.0 persons/km^2^. The climate of the region is humid continental (Köppen climate classification Dfb). Mean elevation of the flat terrain is 106 m, making it easy for the city to expand north and west.

### Multispectral LiDAR data acquisition and data preprocessing

Multispectral Optech Titan LiDAR data were acquired over the study area on 3 September 2014 in a single strip of 3 km^2^ [38,45–47]. The system works with three independent active imaging channels at 1,550 nm (C1), 1,064 nm (C2) and 532 nm (C3). The data were acquired during leaf-on conditions to optimize the geometrical properties of the system, operating at 1075 m above ground with a ±20° scan angle, 200 kHz/channel Pulse Repetition Frequency, and 40-Hz scan frequency [45, 46]. The point clouds were geometrically registered, and LAS files containing xyz coordinates, raw intensity values, the scan angle and the GPS time of each LiDAR point were derived.

In a recent study using Multispectral Optech Titan LiDAR data for land cover classification in Espoo city, near Helsinki on the southern coast of Finland, the authors found that intensity values were not stable across the study area [33]. In our study, we also observed that intensity values were highest in the middle of a flight line and decreased as the distance from the scanner increased. Therefore, to correct this effect, we used a relative radiometric calibration method proposed by [48] as follows:

$$i_{corr}=i*\frac{R_{i}^{2}}{R_{ref}^{2}}$$ Eq 1

where *i*_*corr*_ is the corrected intensity, *i* is the original intensity, R_*i*_ is the distance from the scanner to the scanned point, and R_*ref*_ is the flying altitude (1075 m).

After radiometric calibration of LiDAR intensity data, the lidar point cloud was normalized to height aboveground using lastools [49], and three products were derived and used for land cover classification:

- Mean intensity (IMEAN): we calculated the mean intensity for each channel at grid cell resolution of 1 m for the entire area using lascanopy tool in Lastools [49].
- Pseudo normalized difference vegetation index (PseudoNDVI) [38]:
  $$PseudoNDVI=\frac{\left(IMEAN\;C2-IMEAN\;C3\right)}{\left(IMEAN\;C2+IMEAN\;C3\right)}$$ Eq 2
  where IMEAN C2 and IMEAN C3 are the mean intensity created in the channels 2 and 3 respectively.
- Normalized digital surface model (nDSM): based on the 3D point clouds from the three channels combined, a 1 m nDSM was computed for the entire study area using the canopymodel tool in FUSION/LDV [50].

**Fig 2:**
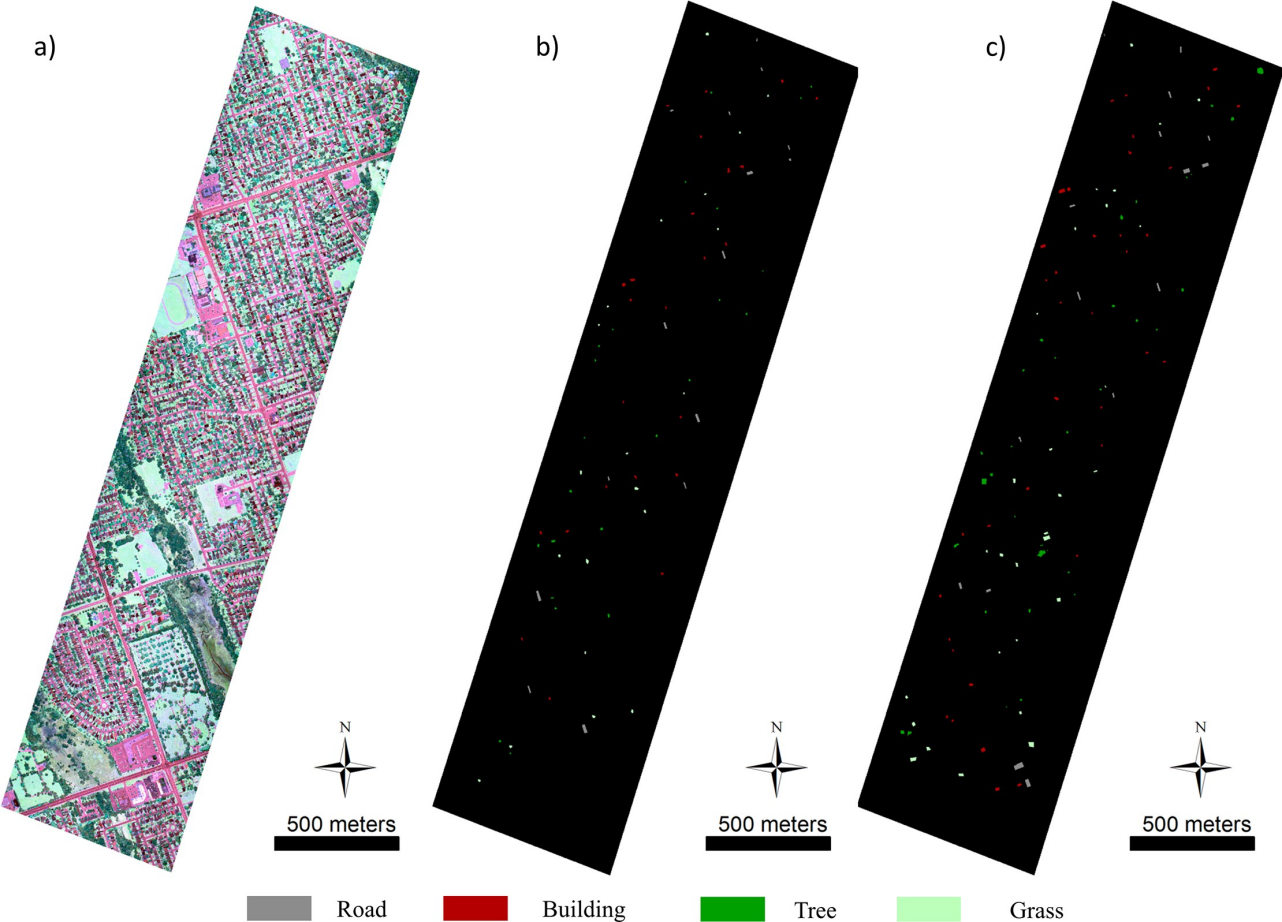
Study area: a) three-band LiDAR intensity color composite image; b) training data; c) validation data.

Four main classes were manually defined based on the intensity image and checked with Google Earth high resolution imagery, including: building, grass, road (including parking places), and trees, which are the most typical land cover types in urban and suburban areas [25, 34]. The three-band LiDAR derived color composite image (C3, C2 and C1 IMEAN bands are separately used as the red, green, and blue) and the corresponding training and validation samples for the study area are shown in Fig 2, and more detailed sample sizes for each class are presented in Table 1.

**Table 1:** Training and validation data sampled from the study area.

	Class	Training Data		Validation Data
1	Road	2,683		3,843
2	Building	2,087		3,850
3	Tree	1,345		3,871
4	Grass	1,655		3,815
	Total	7,770		15,379

### Hierarchical morphological profiles

Herein, we first give a brief introduction on morphological profiles (MP) [44], and then propose the novel Hierarchical Morphological Profiles (HMP).

A MP is composed of the opening profile (OP) and the closing profile (CP). The OP at the pixel x in an image IM is defined as an n-dimensional vector

$${\mathrm{OP}}_{i}\left(x\right)=\gamma_{R}^{\left(i\right)}\left(x\right)\;\forall i\in \left[0,n\right]$$ Eq 3

where $\gamma_{R}^{\left(i\right)}$ is the opening by reconstruction with a SE of size i, and n is the total number of openings. The CP is defined as an n-dimensional vector:

$${\mathrm{CP}}_{i}(x)=\emptyset_{R}^{\left(i\right)}(x)\;\forall i\in [0,n]$$ Eq 4

where $\emptyset_{R}^{\left(i\right)}$ is the closing by reconstruction with a SE of size i. We set CP_0_(x) = OP_0_(x) = IM(x), and the MP of an image I is defined as a 2n+1 –dimensional vector

$$\mathrm{MP}\left(x\right){=\{\mathrm{CP}}_{n}\left(x\right),\ldots ,\mathrm{IM}\left(x\right){,\ldots ,\mathrm{OP}}_{n}\left(x\right)\}$$ Eq 5

The HMP works in the following way: the nDSM data is divided into m layers according to different heights; the image is further split as m layers according to which nDSM layers they belong to; extract the MP features for each layered image; concatenate the extracted MP features, leading to the HMP. In this way, the extracted HMP aims to perform morphological operations in different vertical layers.

**Fig 3:**
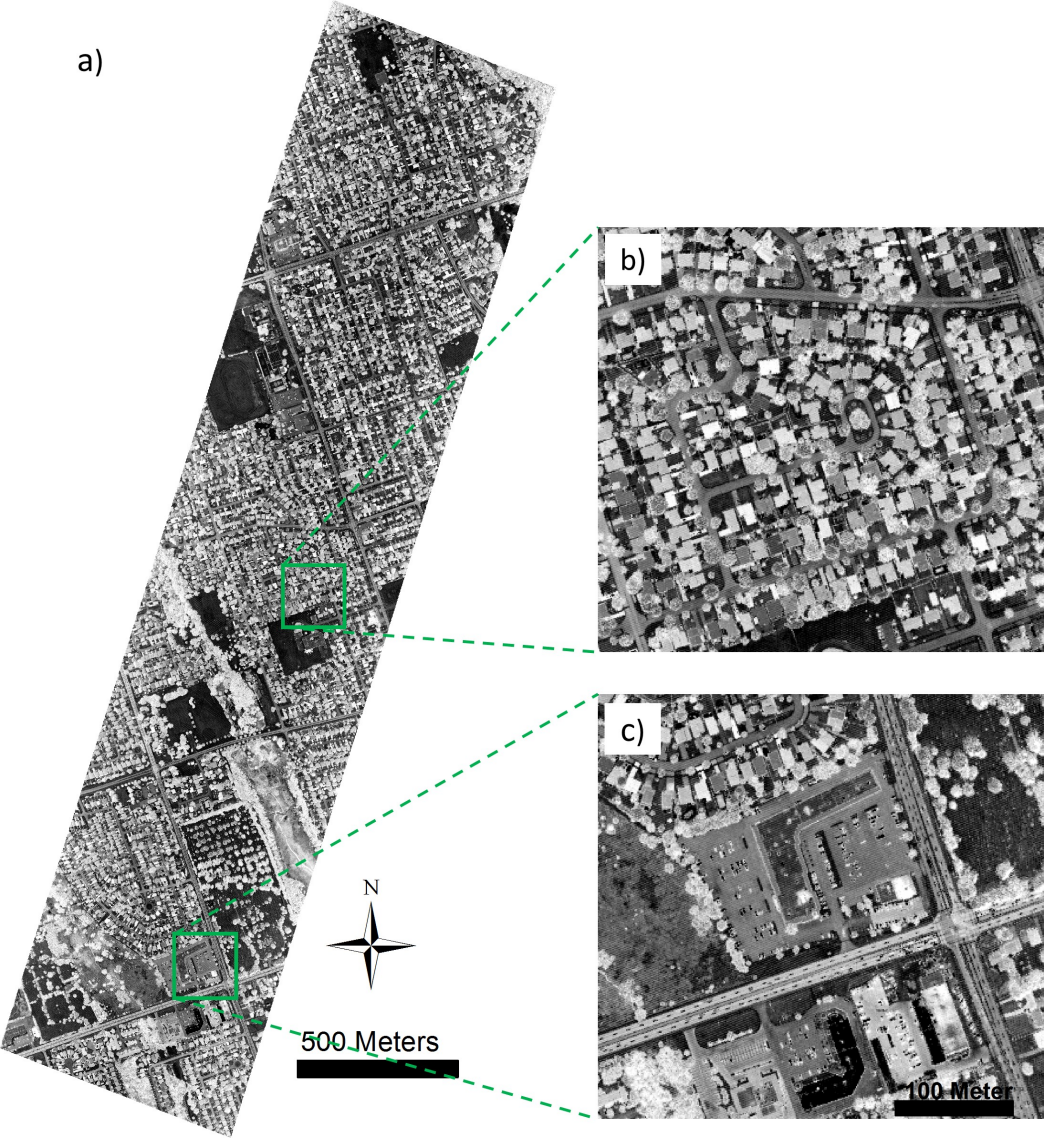
The first principal component of the multispectral LiDAR data in the study area.

Based on the height distribution (i.e., from nDSM data) of the training samples, 3 (i.e., m = 3) height layers are defined: Layer 1: 0 meters = < height < 3 meters; Layer 2: 3 meters = < height < 8 meters; and Layer 3: height > = 8 meters. We applied principal component analysis to the three band intensity image to derive the first principal component (PC) (as shown Fig 3), and used the first PC as characteristic image to extract the MP, with two disk shape SEs empirically set to sizes of 2 and 4, deriving a 5-dimensional MP feature; the first PC was further split into three images based on the height layer, and the MP features were separately extracted and concatenated into a 15-dimensional HMP feature.

### Modeling procedure

There are several ways to combine different features in the classification tasks, for example the composite kernel [51] and multiple kernel learning methods [52]. However, the stacking-vector method is the direct and easiest way to utilize multiple features. Since the main goal is to investigate the usefulness of spatial features in the classification of multispectral LiDAR intensity images, the direct stacking-vector method is adopted to fuse different features.

The support vector machine classifier (using the LIBSVM software package [53]) was adopted as the base classifier due to its good performances for remote sensing image classification [54]. To test the effectiveness of the MP and HMP features individually and in a combined manner with other features, the following models were tested: (i) classification based on the three-band multispectral LiDAR intensity image (IMEAN); (ii) classification based on the stacking vector of multispectral LiDAR intensity image and PseudoNDVI (IMEAN+PseudoNDVI); (iii) classification based on stacking vector of multispectral LiDAR intensity image and nDSM (IMEAN+nDSM); (iv) classification based on stacking vector of multispectral LiDAR intensity image and MP (IMEAN+MP); (v) classification based on stacking vector of multispectral LiDAR intensity image and HMP (IMEAN+HMP); (vi) classification based on stacking vector of multispectral LiDAR intensity image, PseudoNDVI, nDSM and MP (IMEAN+PseudoNDVI+nDSM+MP); and (vii) classification based on stacking vector of multispectral LiDAR intensity image, PseudoNDVI, nDSM and HMP (IMEAN+PseudoNDVI+nDSM+HMP).

For all the models, the radial basis function (RBF) kernel was applied. The value for the regularization parameter C and the gamma value of the RBF kernel were selected by a grid search procedure with five-fold cross validation in the same range {10^−5^,10^−4^,…,10^4^,10^5^}. Classification accuracy was evaluated in terms of overall accuracy (OA, [%]), the Kappa statistic (K), and the class-specific accuracy.

## Results

Classification results are shown in Table 2 for the seven different models tested in the study area. The classification performance based on the three-band intensity image is relatively moderate (74.66%). However, auxiliary features extracted from the intensity image were found very useful for improving the classification accuracy. Among the extracted features, PseudoNDVI, nDSM, MP, and HMP separately improve 0.37%, 12.12%, 7.65%, and 18.14% in terms of overall accuracy compared to the intensity image. When combining the intensity image with PseudoNDVI, nDSM, and HMP features, the overall classification accuracy increases by 18.62%.

**Table 2:** Accuracy (%) of different classification models in the study area.

	IMEAN	IMEAN + PseudoNDVI	IMEAN+nDSM	IMEAN+ MP	IMEAN+ HMP	IMEAN+PseudoNDVI +nDSM+MP	IMEAN+PseudoNDVI+nDSM+HMP
							
Road	76.66	75.98	95.81	87.69	98.28	98.23	98.56
Building	42.42	45.01	80.44	50.13	93.01	77.95	91.90
Tree	93.85	93.07	82.46	95.94	84.86	92.12	87.19
Grass	85.71	85.82	88.49	95.54	95.10	95.81	95.51
							
OA	74.66	74.97	86.78	82.31	92.80	91.01	93.28
Kappa	0.54	0.67	0.82	0.76	0.90	0.88	0.91

As for the specific classes, class 1 (road) and class 2 (building) have the worst classification accuracy (76.66% and 42.42%) based on the intensity image, due to confusion between these classes (see the confusion matrix Table 3). While the nDSM and MP features separately improve the classification, the proposed HMP feature greatly improves the classification accuracy for these two classes. On combing all the features (i.e., IMEAN+PseudoNDVI+nDSM+HMP), the best classification model is achieved (with highest overall accuracy and Kappa coefficient, also, see the confusion matrix Table 4; confusion matrices (S1–S5 Tables) for the other models are provided in the Supplementary Materials).

**Table 3:** Confusion matrix for the IMEAN classification model.

		**Reference Data**					
		Road	Building	Tree	Grass	Total	User’s Accuracy
**Predicted Data**	Road	2,946	1,325	102	354	4,727	62.32%
	Building	743	1,633	96	20	2,492	65.53%
	Tree	56	629	3,633	171	4,489	80.93%
	Grass	98	263	40	3,270	3,671	89.08%
	Total	3,843	3,850	3,871	3,815	15,379	
	Producer’s Accuracy	76.66%	42.42%	93.85%	85.71%		
** Overall Accuracy: 74.66%; Kappa: 0.54**							

Correctly classified pixels are highlighted in grey.

**Table 4:** Confusion matrix for the (IMEAN+nDSM+PseudoNDVI+HMP) classification model.

		**Reference Data**					
		Road	Building	Tree	Grass	Total	User’s Accuracy
**Predicted Data**	Road	3,788	22	25	162	3,997	94.77%
	Building	19	3,538	317	2	3,876	91.28%
	Tree	14	219	3,375	7	3,615	93.36%
	Grass	22	71	154	3,644	3,891	93.65%
	Total	3,843	3,850	3,871	3,815	15,379	
	Producer’s Accuracy	98.57%	91.90%	87.19%	95.52%		
** Overall Accuracy: 93.28%; Kappa: 0.91**							

Correctly classified pixels are highlighted in grey.

**Fig 4:**
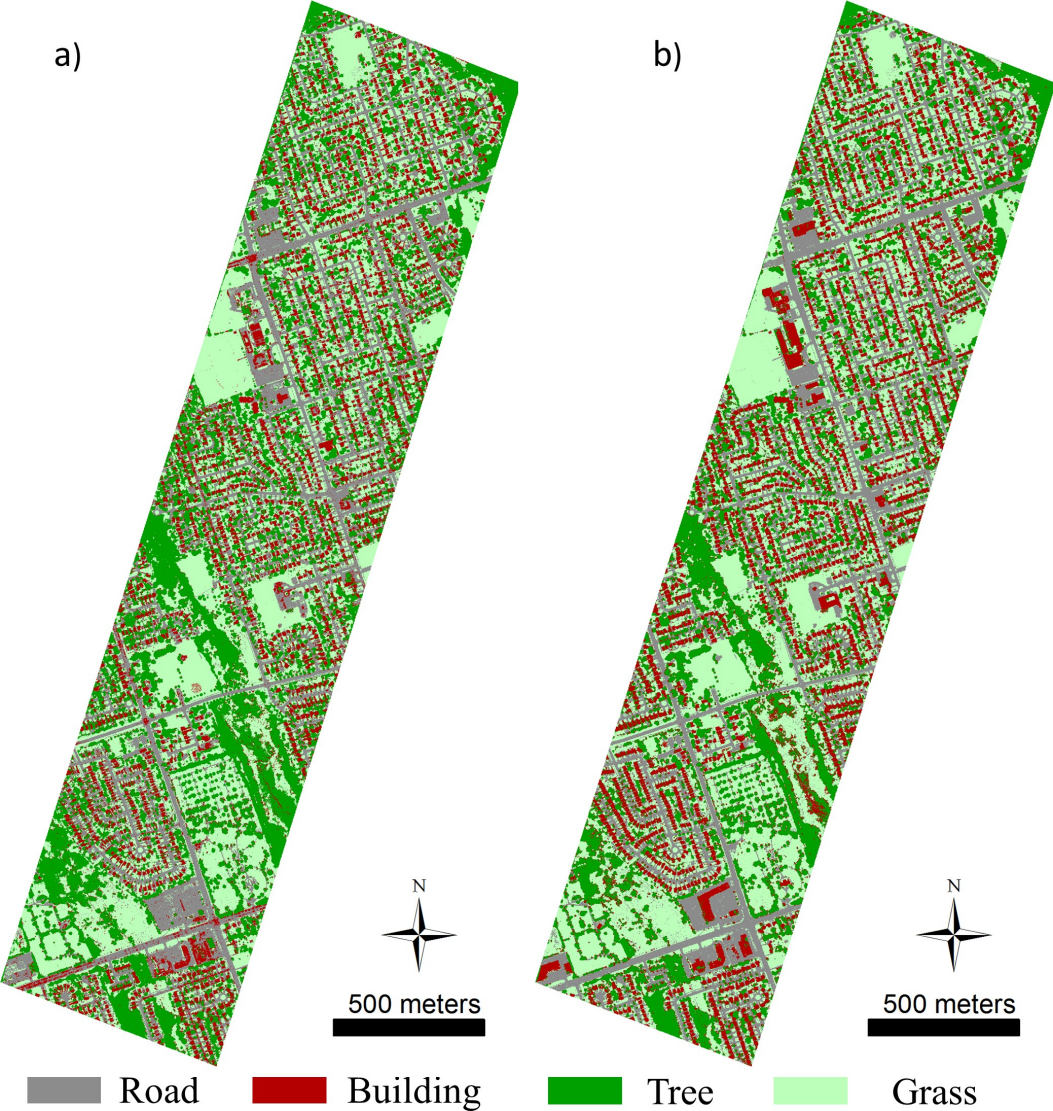
Classification maps using a) IMEAN model and b) IMEAN+PseudoNDVI+nDSM+HMP model.

Classification maps for the IMEAN and IMEAN+PseudoNDVI+nDSM+HMP are shown in Fig 4 (the classification maps (S1–S3 Figs) for other tested models are presented in the Supplementary Materials). The classification map for the intensity image has the most noise due to many misclassifications, while the second map (the one combining mean intensity data, PseudoNDVI, nDSM, and MP) greatly reduces the classification errors, showing a more spatially consistent classification result.

**Fig 5:**
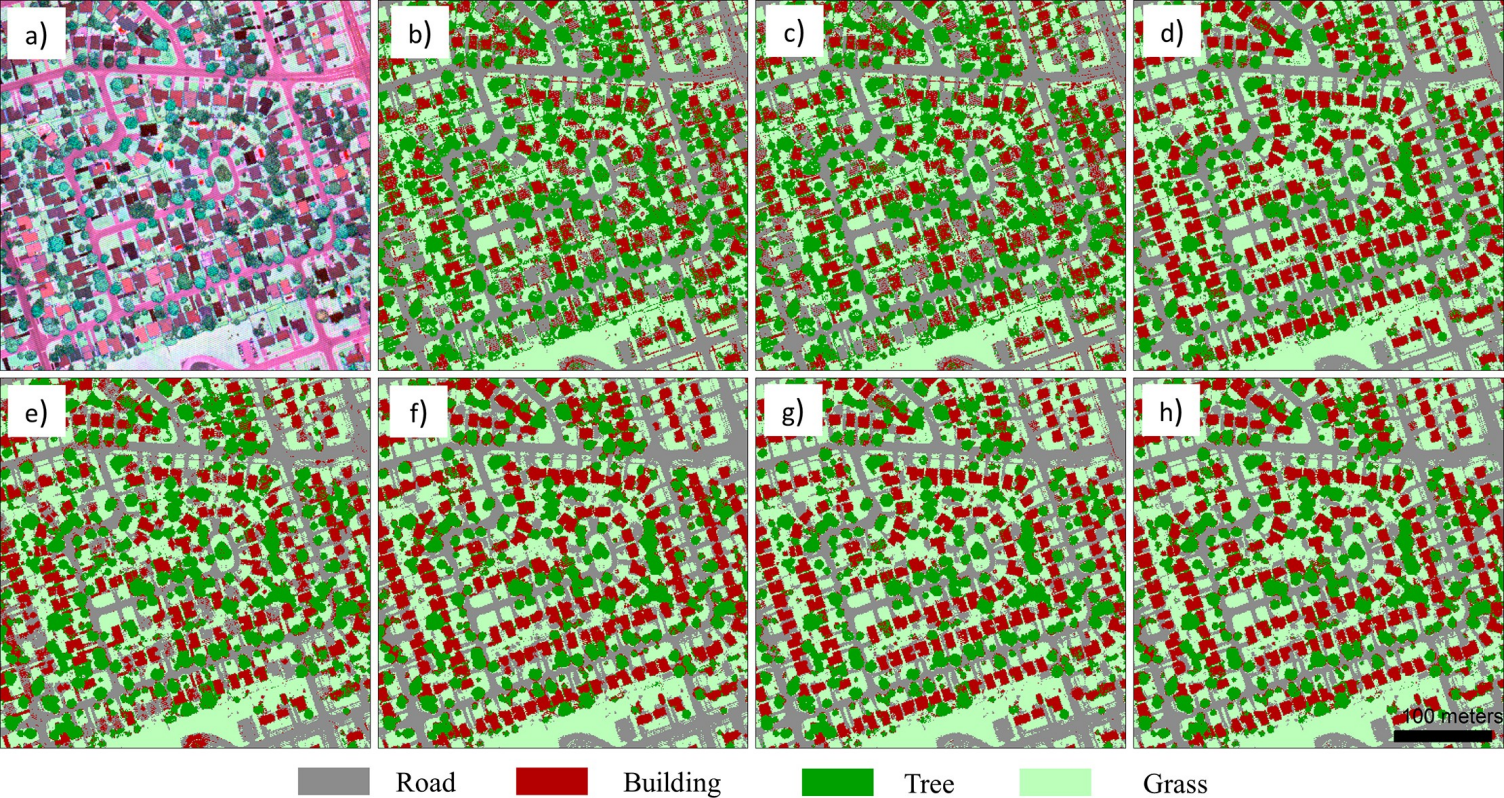
Zoomed-in views of subset area 1: a) LiDAR intensity color composite image; classification maps for the b) IMEAN model; c) IMEAN+PseudoNDVI model; d) IMEAN+nDSM model; e) IMEAN+MP model; f) IMEAN+HMP model; g) IMEAN+PseudoNDVI+nDSM+MP model; and h) IMEAN+PseudoNDVI+nDSM+HMP model.

**Fig 6:**
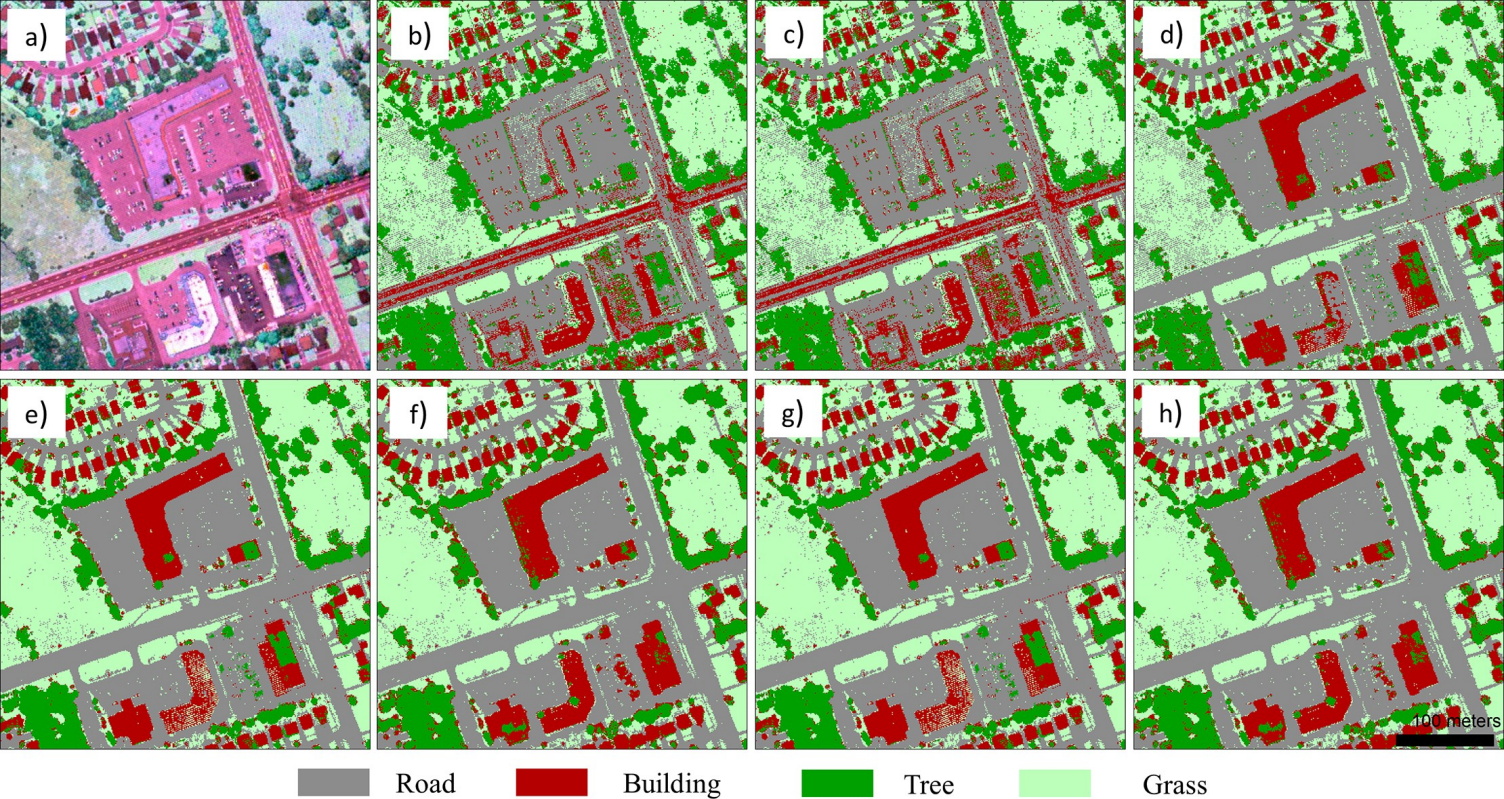
Zoomed-in views of subset area 2: a) LiDAR intensity color composite image; classification maps for the b) IMEAN model; c) IMEAN+PseudoNDVI model; d) IMEAN+nDSM model; e) IMEAN+MP model; f) IMEAN+HMP model; g) IMEAN+PseudoNDVI+nDSM+MP model; and h) IMEAN+PseudoNDVI+nDSM+HMP model.

To illustrate better map detail, zoomed-in subsets of two typical areas (i.e., residential area, and commercial area) within the urban area are separately shown in Figs 5 and 6. The high accuracy building map in Fig 5 and building and road map in Fig 6 clearly show that the proposed HMP features can greatly improve the classification results.

## Discussion

### Importance of spatial features for multispectral LiDAR classification

In this rapidly changing world, timely and accurate classification of urban areas is crucial for urban planning and sustainable management [1, 55]. In this study, we are making use of cutting-edge multispectral LiDAR sensor (Optech Titan) technology and auxiliary information derived from the multispectral LiDAR for land cover classification; by combining multispectral LiDAR data and morphological profiles we classify terrains in urban areas. The classification results from our study demonstrate the capability of multispectral LiDAR data in recording a diversity of spectral signals from land cover objects and thereby underscores its potential for effective urban area classification.

Herein, we classified the multispectral LiDAR intensity data into four classes, namely buildings, trees, roads, and grass, and obtained highest overall classification accuracies when a combination of intensity data, PseudoNDVI, nDSM, and HMP was used. Previous studies have shown that multispectral LiDAR data provides more benefits for urban land cover classification [29]; to improve classification accuracy, PseudoNDVI and DSM or nDSM features have been utilized [34, 37, 56–58]. Consistent with previous studies, this study further demonstrated that PseudoNDVI and nDSM can improve classification accuracy of a multispectral LiDAR intensity image.

In addition, this study further demonstrated that inclusion of spatial features (MP and the proposed HMP) is markedly useful for deriving high accuracy classification results compared to methods employing only multispectral LiDAR intensity data; specifically, the road and building classes benefited the most in our case. In general, it is hard to classify the aforementioned classes due to multiple reasons; roads in the urban areas are filled with vehicles, thus resulting in highly reflective spots in the image (see Fig 6A); buildings are usually covered with different colored roofs (see Fig 5A and Fig 6A). Although the nDSM feature brings greatly improved classification accuracy for these two classes due to their distinct height distributions, it also introduced more classification errors between trees and grass (see the confusion matrix S2 Table), thus lowering the classification accuracies for these two classes. MP improved the classification accuracy of roads and buildings by 11.03% and 7.71%, respectively. However, many classification errors between these two classes resulted by including the MP feature (e.g., producer accuracy for the building class is only up to 50.13%), because it lacks the ability to operate in the vertical dimension. The proposed HMP considers the height information by extracting the MP feature over different vertical layers. In this way, the classification accuracy improved for these two classes by up to 98.23% and 93.01%. This demonstrates that the MP feature is useful for classification of multispectral LiDAR intensity data by capturing the spatial characteristics of different land cover types. By incorporating the vertical information (usually provided by the nDSM data from the LiDAR data), the proposed HMP feature provided better discrimination than the MP feature.

### Comparisons with previous studies

The support vector machines classifier was adopted as the base classifier for this study due to its robustness and high accuracy for remote sensing image classification [54, 59, 60]. Stacking vectors of HMP with the multispectral LiDAR intensity image was found to increase classification accuracy significantly, resulting in an overall accuracy of 93.28% for the study area. Alternative methods that have been used for classification related to multispectral LiDAR data are object-based analysis, data clustering methods, and maximum likelihood classifiers. In Zou et al. [37], an Object Based Image Analysis (OBIA) approach for 3D land cover classification using multispectral LiDAR point clouds was presented; an overall accuracy of over 90% was obtained while classifying the landcover types into 9 categories. However, some misclassified objects–such as roads classified as lawn and bare soil–resulted due to similar spectral properties and at times because of lack of effective spectral and spatial features for distinguishing class boundaries. Matikainen et al. [33] evaluated the use of different spectral indices derived from multispectral LiDAR data for land cover classification and map updating; the classes considered for the study included building, tree, asphalt, gravel, rocky area and low vegetation, and obtained an accuracy of 96% compared with validation points. Multispectral Lidar data combined with old map vectors proved to enhance automated change detection of buildings, and assisted in removal of shadows on intensity images produced from the data; the passive aerial imaging commonly used in mapping suffered from external illumination conditions and often resulted in excessive shadows on intensity images. Bakula et al. [35] fused multi-wavelength laser intensity imagery, elevation data and textural information, and applied spectral (using maximum likelihood rule) and spectral-textural classification approaches for distinguishing 6 classes; they got an overall accuracy of 90%. In that study, applications of additional morphological classification and granulometric transformations were found to highly enhance the accuracy of the separation of building and road classes, as they eliminated several pixels that were initially confused. They also noticed that interpolating the intensity raster was not very helpful for improving classification results; even though using intensity rasters with both first and last returns slightly benefited the study. In essence, our strategy of using intensity features for classification resulted in accuracies similar to related studies, and considering the boost it gave to the overall classification (18.62%) obtained through the combination of intensity image with PseudoNDVI, nDSM, and HMP features, this method may be efficient for future multispectral LiDAR endeavors; e.g., plant species classification [61–63], urban change detection [64,65], flood inundation mapping [66] and even carbon sequestration modeling [67].

### Implications and future directions

Although multispectral LiDAR data can be considered a pivotal tool for bolstering subsequent urban planning and mapping operations [36, 38, 68], data processing should be undertaken with caution. It should be borne in mind that several challenges—associated with selection of suitable geometric and radiometric correction equations for large spatial extents, fitting classification methods when the number of classes is large, appropriate interpolation techniques for creating the intensity raster, range ambiguities during data collection, intensity heterogeneity and energy loss (primarily caused due to narrow scan angle), and unidentified influences of laser beam incident angles and illumination of the target material on LiDAR intensity data–already exist, and these issues need to be addressed for fruitful exploitation of multispectral data [35–37, 69]. In addition, the towering cost associated with multispectral LiDAR sensors limits its applicability. Therefore, before acquiring the multispectral LiDAR, the purpose and agenda should be well defined to ensure that the results will justify the investment and meet expectations. In this regard, we recommend the use of multispectral LiDAR on a case-by-case basis, and operations such as land cover classification should be considered as a byproduct or one of the multiple objectives, while using multispectral LiDAR for optimizing the amount spent on data acquisition.

## Conclusion

In this study, we assessed the capability of a cutting-edge LiDAR sensor, Multispectral Optech Titan, combined with advanced modelling derivatives, for classifying land cover in an urban area. Specifically, we only considered the raster products from LiDAR data (i.e., intensity data and nDSM data), and extracted the MP features from intensity data. A novel hierarchical morphological profiles feature was proposed to extract spatial features of multispectral LiDAR intensity data while maintaining vertical structure information. Results show that the MP feature is useful for providing spatially consistent land cover classification in urban areas. In addition, the proposed HMP feature was found to work best among different features for the multispectral LiDAR data by making use of height information. We obtained an overall accuracy of 93.28% for land cover classification of four classes in the urban area from our best tested model (IMEAN+PseudoNDVI+nDSM+HMP). The results from ourstudy demonstrate that classification results can be greatly improved by extracting spatial features from three-band LiDAR intensity composite images. Future studies could further exploit spectral-spatial classification methods applied to multispectral LiDAR data, and possibly directly classify the point cloud data (i.e., considering geometrical features), which poses new challenges for feature extraction methods.

## Supporting information

S1 Fig Classification maps for the a) IMEAN+PseudoNDVI model and b) IMEAN+nDSM model.
(TIF)

S2 Fig Classification maps for the a) IMEAN+MP model and b) IMEAN+HMP model.
(TIF)

S3 Fig Classification map for the IMEAN+PseudoNDVI+nDSM+MP model. (TIF)

S1 Table Confusion matrix for the IMEAN+PseudoNDVI classification model. Correctly classified pixels are highlighted in grey.
(PDF)

S2 Table Confusion matrix for the IMEAN+nDSM classification model. Correctly pixels are highlighted in grey.
(PDF)

S3 Table Confusion matrix for the IMEAN+MP classification model. Correctly classified pixels are highlighted in grey.
(PDF)

S4 Table Confusion matrix for the IMEAN+HMP classification model. Correctly pixels are highlighted in grey.
(PDF)

S5 Table Confusion matrix for the IMEAN+PseudoNDVI+nDSM+MP classification model. Correctly classified pixels are highlighted in grey.
(PDF)
