# Supplementary figures and images for: Supervised spatial classification of multispectral LiDAR data in urban areas
Source: PLoS One. 2018 Oct 24;13(10):e0206185. doi: 10.1371/journal.pone.0206185 (PMC6200265; doi:10.1371/journal.pone.0206185)

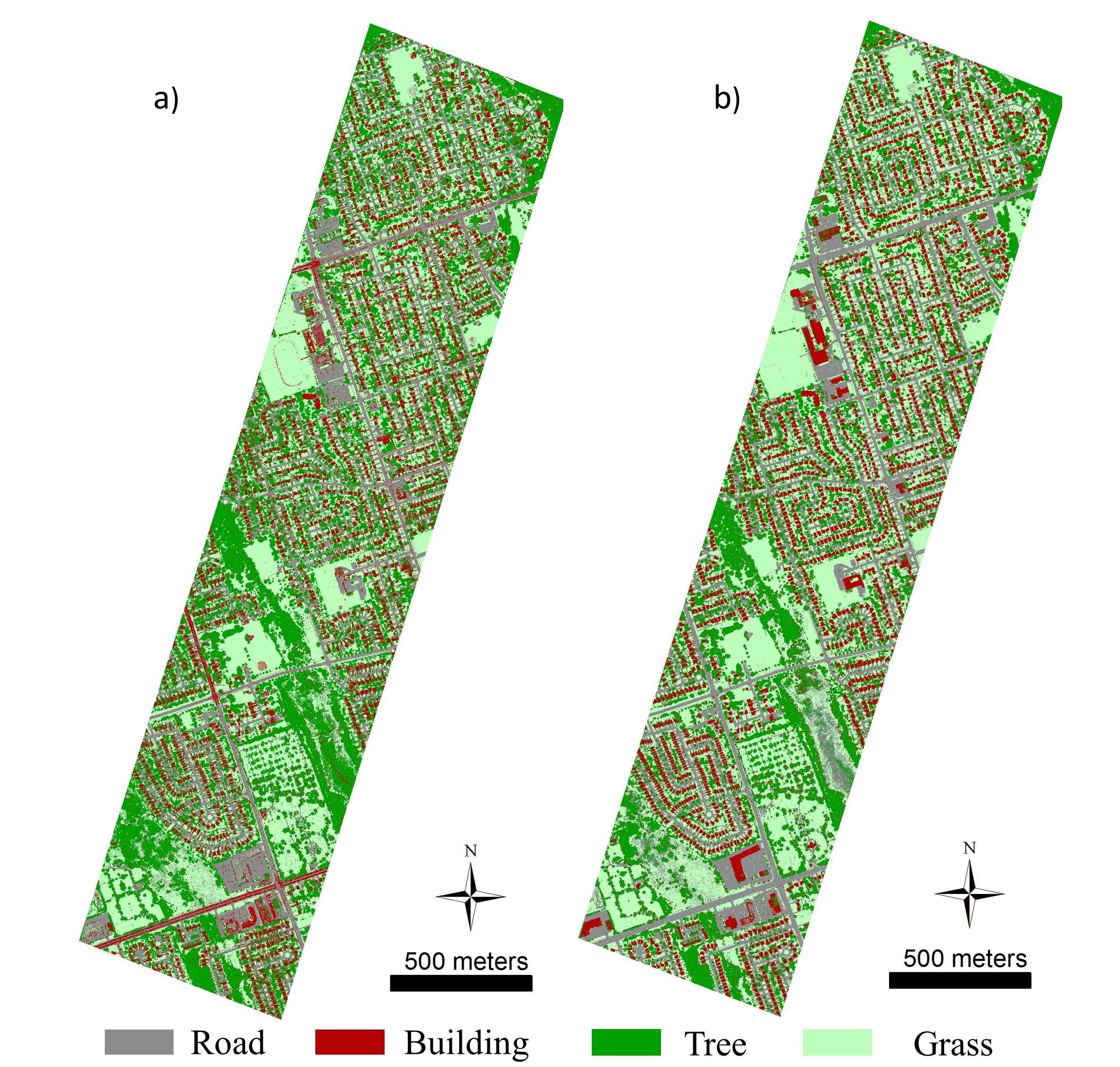

Supplement: S1 Fig — Classification maps for the a) IMEAN+PseudoNDVI model and b) IMEAN+nDSM model. (TIF) [file pone.0206185.s001.tif]

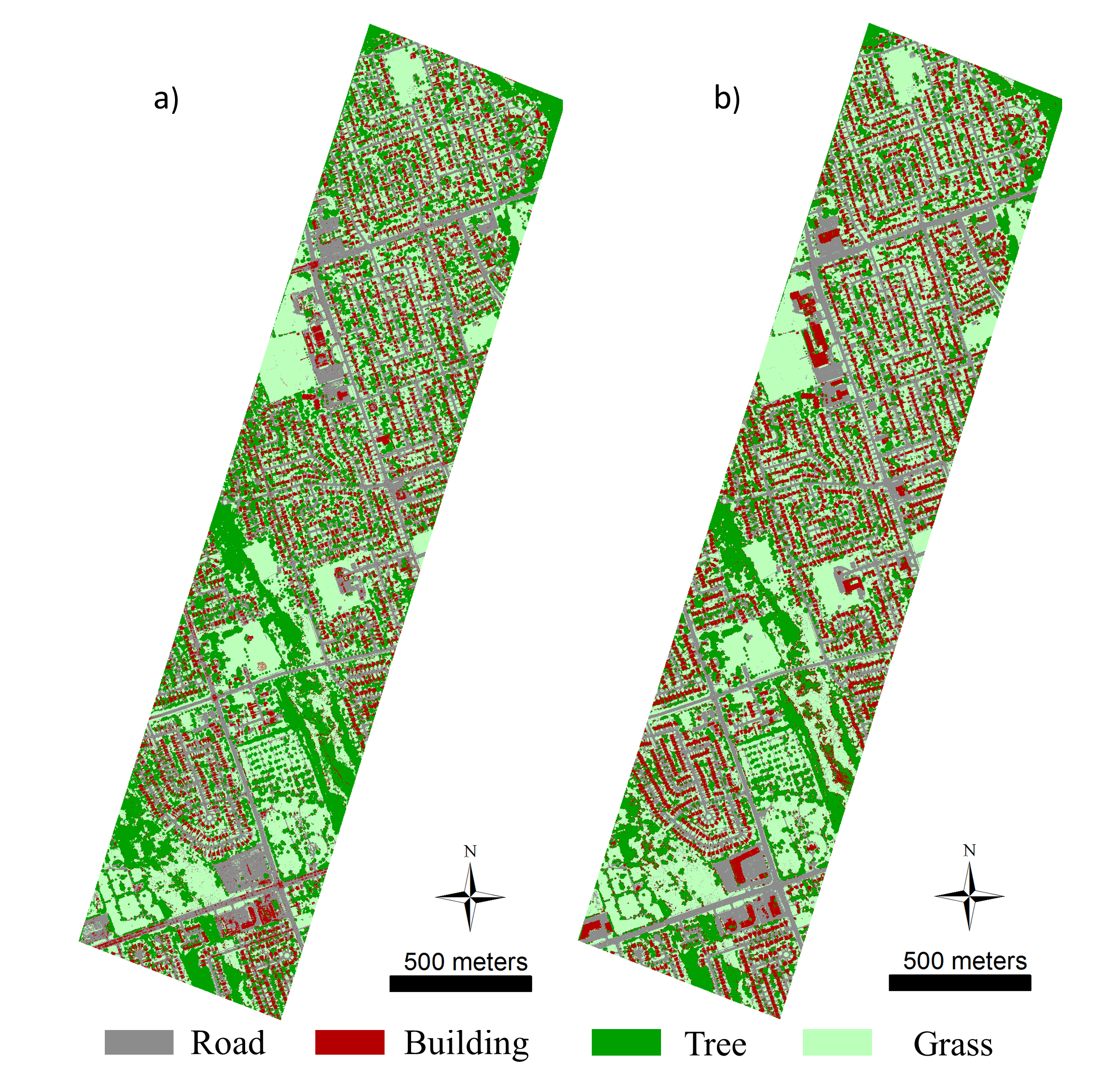

Supplement: S2 Fig — Classification maps for the a) IMEAN+MP model and b) IMEAN+HMP model. (TIF) [file pone.0206185.s002.tif]

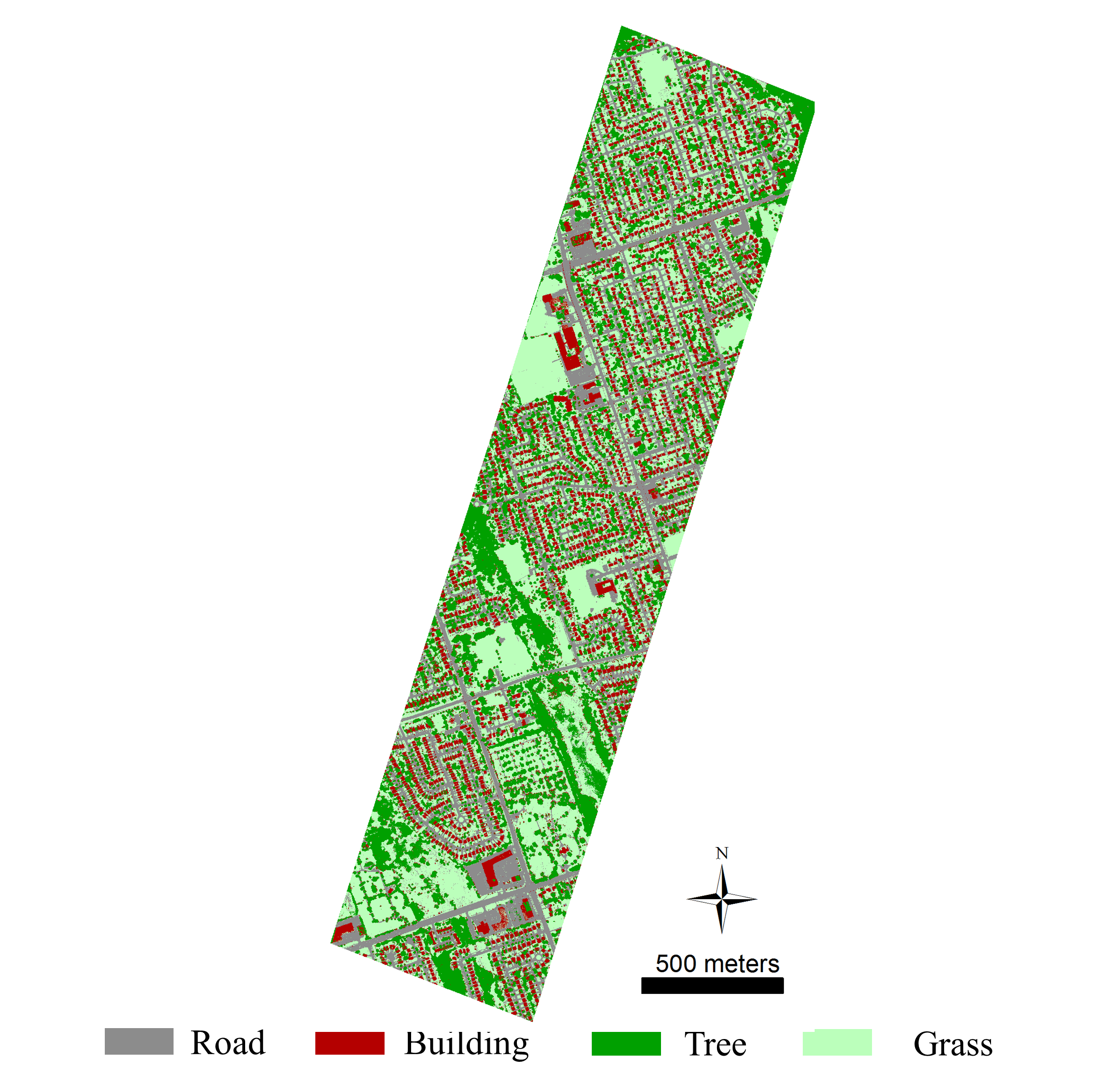

Supplement: S3 Fig — (TIF) [file pone.0206185.s003.tif]
