# Supplementary material for: Supervised spatial classification of multispectral LiDAR data in urban areas
Source: PLoS One. 2018 Oct 24;13(10):e0206185. doi: 10.1371/journal.pone.0206185 (PMC6200265; doi:10.1371/journal.pone.0206185)
Supplement: S1 Table — Correctly classified pixels are highlighted in grey. (PDF) [file pone.0206185.s004.pdf]

S1 Table. Confusion matrix for the IMEAN+PseudoNDVI classification model. Correctly classified pixels are highlighted in grey.

		Reference Data					
		Road	Building	Tree	Grass	Total	User's Accuracy
**Predicted Data**	Road	2,920	1,256	85	323	4,584	63.70%
	Building	782	1,733	138	24	2,677	64.74%
	Tree	50	599	3,603	194	4,446	81.04%
	Grass	91	262	45	3,274	3,674	89.11%
	Total	3,843	3,850	3,871	3,815	15,379	
	Producer's Accuracy	75.98%	45.01%	93.07%	85.82%		

Overall Accuracy: 74.97%; Kappa: 0.67
