# Supplementary material for: Supervised spatial classification of multispectral LiDAR data in urban areas
Source: PLoS One. 2018 Oct 24;13(10):e0206185. doi: 10.1371/journal.pone.0206185 (PMC6200265; doi:10.1371/journal.pone.0206185)
Supplement: S2 Table — Correctly pixels are highlighted in grey. (PDF) [file pone.0206185.s005.pdf]

S2 Table. Confusion matrix for the IMEAN+nDSM classification model. Correctly pixels are highlighted in grey.

		Reference Data					
		Road	Building	Tree	Grass	Total	User's Accuracy
Predicted Data	Road	3,682	215	372	414	4,683	78.62%
	Building	37	3,097	201	4	3,339	92.75%
	Tree	24	424	3,192	24	3,661	87.19%
	Grass	100	114	106	3,376	3,696	91.34%
	Total	3,843	3,850	3,871	3,815	15,379	
	Producer's Accuracy	95.81%	80.44%	82.46%	88.49%		

Overall Accuracy: 86.78%; Kappa: 0.82
