# Supplementary material for: Supervised spatial classification of multispectral LiDAR data in urban areas
Source: PLoS One. 2018 Oct 24;13(10):e0206185. doi: 10.1371/journal.pone.0206185 (PMC6200265; doi:10.1371/journal.pone.0206185)
Supplement: S4 Table — Correctly pixels are highlighted in grey. (PDF) [file pone.0206185.s007.pdf]

S4 Table. Confusion matrix for the IMEAN+HMP classification model. Correctly pixels are highlighted in grey.

		Reference Data					
		Road	Building	Tree	Grass	Total	User's Accuracy
Predicted Data	Road	3,777	19	29	160	3,985	94.78%
	Building	22	3,581	465	1	4,069	88.01%
	Tree	12	164	3,285	26	3,487	94.21%
	Grass	32	86	92	3,628	3,838	94.53%
	Total	3,843	3,850	3,871	3,815	15,379	
	Producer's Accuracy	98.23%	93.01%	84.86%	95.10%		

Overall Accuracy: 92.80%; Kappa: 0.90
