# Supplementary material for: Supervised spatial classification of multispectral LiDAR data in urban areas
Source: PLoS One. 2018 Oct 24;13(10):e0206185. doi: 10.1371/journal.pone.0206185 (PMC6200265; doi:10.1371/journal.pone.0206185)
Supplement: S3 Table — Correctly classified pixels are highlighted in grey. (PDF) [file pone.0206185.s006.pdf]

S3 Table. Confusion matrix for the IMEAN+MP classification model. Correctly classified pixels are highlighted in grey.

		Reference Data					
		Road	Building	Tree	Grass	Total	User's Accuracy
**Predicted Data**	Road	3,370	974	22	119	4,485	75.14%
	Building	433	1,930	102	34	2,499	77.23%
	Tree	6	630	3,714	17	4,367	85.05%
	Grass	34	316	33	3,645	4,028	90.49%
	Total	3,843	3,850	3,871	3,815	15,379	
	Producer's Accuracy	87.69%	50.13%	95.94%	95.54%		

Overall Accuracy: 82.31%; Kappa: 0.76
