# Supplementary material for: Supervised spatial classification of multispectral LiDAR data in urban areas
Source: PLoS One. 2018 Oct 24;13(10):e0206185. doi: 10.1371/journal.pone.0206185 (PMC6200265; doi:10.1371/journal.pone.0206185)
Supplement: S5 Table — Correctly classified pixels are highlighted in grey. (PDF) [file pone.0206185.s008.pdf]

S5 Table. Confusion matrix for the IMEAN+PseudoNDVI+nDSM+MP classification model. Correctly classified pixels are highlighted in grey.

		**Reference Data**					
		Road	Building	Tree	Grass	Total	User's Accuracy
**Predicted Data**	Road	3,775	43	27	155	4,000	94.38%
	Building	42	3,001	144	5	3,192	94.02%
	Tree	5	535	3,566	0	4,106	86.85%
	Grass	21	271	134	3,655	4,081	89.56%
	Total	3,843	3,850	3,871	3,815	15,379	
	Producer's Accuracy	98.23%	77.95%	92.12%	95.81%		

Overall Accuracy: 91.01%; Kappa: 0.88
